# Editorial: Sarcoidosis and autoimmunity: From bench to bedside

**DOI:** 10.3389/fmed.2023.1147529

**Published:** 2023-02-21

**Authors:** Miriana d'Alessandro

**Affiliations:** Respiratory Diseases Unit, Department of Medical and Surgical Sciences & Neurosciences, University of Siena, Siena, Italy

**Keywords:** sarcoidosis, autoimmunity, biomarkers, personalized medicine, granuloma

Sarcoidosis is a granulomatous disease of unknown origin with multisystem involvement. It mainly affects the lungs and intrathoracic lymph nodes, featuring noncaseating granulomas (1). The clinical course is variable, and there is no universally accepted treatment algorithm (2). Its prevalence depends on geographical distribution (3), ranging from 1 to 5 per 100,000 in South Korea (4), Taiwan (5), and Japan (6) to 140–160 per 100,000 in Sweden (7) and Canada (8).

The diagnosis of sarcoidosis is formulated by multidisciplinary evaluation of clinical, radiological, and immunological findings (9), especially those of organs seldom affected such as the kidneys, liver, and heart, which are underdiagnosed yet clinically significant forms with significant morbidity and mortality. It has been reported that 25–43% of patients with sarcoidosis have clinically silent renal involvement (10). To detect early kidney involvement and begin treatment immediately, Calatroni et al. recommended systematic screening of renal function, including serum creatinine, urine analysis, and serum calcium levels. A kidney transplant is considered an acceptable option for patients with uremic sarcoidosis, and complications seem to be similar to those associated with a kidney transplant for other types of renal failure.

No specific and sufficiently accurate biomarkers have yet been identified for the clinical management of sarcoidosis. Among diagnostic markers, angiotensin-converting enzyme (ACE) (11) has a controversial role in diagnosing or managing sarcoidosis as it is not sensitive enough for the diagnosis of systemic sarcoidosis: the rate of false positives is high, and 50% of patients with sarcoidosis show normal levels at disease onset. Sedki et al. suggest lung imaging and measurement of serum ACE concentrations at the initial evaluation of sarcoidosis. However, further investigation is warranted in patients with intrahepatic cholestasis, negative for anti-mitochondrial antibodies, and with liver biopsy evidence of granulomas, especially if these are predominantly lobular. Sedki et al. recommend a primary bile cirrhosis-specific anti-nuclear antibody (ANA) (gp210 and sp100) test in the case of ANA positivity.

A major complication of hepatic sarcoidosis is portal hypertension (PH), a prognostic marker associated with high morbidity and mortality. Fauter et al. analyzed this complication in 12 patients with histological evidence of hepatic sarcoidosis. They highlighted the ineffectiveness of PH management and/or sarcoidosis therapy in these patients, concluding that a liver transplant could be the best therapeutic option when corticosteroids fail.

Despite extensive research, the etiology and pathogenesis of sarcoidosis remain largely unknown. Several triggers have been implicated in its development, including autoantigen-specific T cells, antibodies producing B lymphocytes, and autoimmune inflammation. Sarcoidosis is not classified as an autoimmune disorder, although autoimmune components play an important role in its pathogenesis.

Rizzi et al. reviewed the relationship between sarcoidosis and autoimmunity, highlighting the role of humoral immunity in its pathogenesis. Although patients with sarcoidosis are inclined to develop specific autoantibodies, this is postulated to occur by molecular mimicry. In susceptible individuals, it occurs when there is a similarity between a foreign and a self-peptide, which promotes the activation of autoreactive T and B cells. Clinical improvement with anti-CD20 monoclonal antibody therapy, observed in some refractory cases of sarcoidosis, supports this argument. B-cell depletion has provided insights into the importance of autoimmunity in this immunological context. Several pathogenetic pathways and genetic predispositions are common to autoimmune diseases. The review article of Malkova et al. describes a genetic predisposition to chronic progressive sarcoidosis that prevents antigen elimination and promotes autoimmune inflammation, impairing the immune system or activating autoimmune disorders. Fibrosis is associated with fibroblast activation after the differentiation of leukocytes which release cytokines in a setting of chronic inflammation.

Irrespective of organ involvement, the etiopathogenesis of fibrosis and failure of affected organs in patients with progressive sarcoidosis is still debated. Patterson et al. suggested that failure to clear antigens contributes to cardiac sarcoidosis and that the distribution of lymph node activity in the thoracic region differs over time. According to the study, lymph nodes are more likely to be involved in the progression of sarcoidosis in patients with chronic disease.

It has been reported that lymph nodes are often involved in sarcoidosis, and a number of studies have been carried out using samples obtained by endobronchial ultrasound-guided transbronchial needle aspiration (EBUS-TBNA) to explore the pathogenesis and establish diagnostic biomarkers. Zhao et al. performed genome-wide miRNA profiling in the lymph nodes of patients with sarcoidosis, comparing it with the profiles of patients with tuberculosis lymphadenitis (TBLN) and demonstrating the high diagnostic value of such profiles for sarcoidosis. MiR-185-5p is still useful in the differential diagnosis of TBLN and sarcoidosis, particularly when patients with the latter disease are in stage I or II.

The purpose of this Research Topic is to present some new experimental findings and updated reviews on organs rarely affected by sarcoidosis and the relationship between autoimmune diseases and sarcoidosis. At the threshold of a new era of personalized treatment, the discovery of new therapeutic targets could help prevent the development of chronic inflammation and tissue damage in these patients.

## Author contributions

The author confirms being the sole contributor of this work and has approved it for publication.
